# The Oncogenic Role of Long Non-Coding RNA *NEAT1* in Head and Neck Squamous Cell Carcinoma: From Molecular Mechanisms to Clinical Implications

**DOI:** 10.3390/biology15040307

**Published:** 2026-02-10

**Authors:** Yuanxin Shi, Bin Chen, Guohui Bai

**Affiliations:** Key Laboratory of Oral Disease Research, School of Stomatology, Zunyi Medical University, Zunyi 563000, China; shiyuanxin@zmu.edu.cn (Y.S.); chenbin@zmu.edu.cn (B.C.)

**Keywords:** biomarker, prognosis, diagnosis, long non-coding RNA NEAT1, ceRNA network

## Abstract

Head and neck squamous cell carcinoma (HNSCC) is an aggressive malignancy with poor patient outcomes. The long non-coding RNA *NEAT1* (lncRNA *NEAT1*) has emerged as a critical driver of HNSCC pathogenesis. This review synthesizes current knowledge on lncRNA *NEAT1*’s aberrant expression, molecular mechanisms, and functional roles in HNSCC. LncRNA *NEAT1* is significantly upregulated in tumors and promotes progression by acting as a competing endogenous RNA (ceRNA) for multiple miRNAs, such as miR-125b-5p, miR-204, and miR-34a-5p, thereby regulating downstream targets including *SLC1A5*, *ZEB1*, and components of the Wnt/β-catenin pathway. These interactions drive key oncogenic processes, including proliferation, metastasis, epithelial–mesenchymal transition, therapy resistance, and cell death inhibition. Clinically, high lncRNA *NEAT1* expression correlates with advanced T stage, lymph node metastasis, and reduced survival, underscoring its potential as a diagnostic and prognostic biomarker. Therapeutically, emerging approaches such as nanoparticle-mediated delivery of siRNA/shRNA offer a promising strategy to target lncRNA *NEAT1*, potentially synergizing with existing immunotherapies. Although clinical translation remains challenging, lncRNA *NEAT1* represents a highly promising biological target for future precision oncology in HNSCC.

## Simple Summary

Head and neck squamous cell carcinoma (HNSCC) is an aggressive cancer with limited treatment options and poor survival rates. This review focuses on a specific long non-coding RNA *NEAT1* (LncRNA *NEAT1*), which is often found at high levels in HNSCC. We summarize how LncRNA *NEAT1* acts as a key driver of cancer progression—promoting tumor growth, spread, and resistance to therapy—by interacting with multiple microRNAs and activating several cancer-related signaling pathways. Clinically, high LncRNA *NEAT1* expression is linked to advanced disease stage, metastasis, and worse patient outcomes, indicating its potential as a diagnostic and prognostic biomarker. Although directly targeting LncRNA *NEAT1* therapeutically remains challenging, emerging approaches such as nanoparticle-based gene silencing offer promising strategies. Understanding the role of LncRNA *NEAT1* may help advance precision medicine and improve future treatments for HNSCC patients.

## 1. Introduction

Head and Neck Squamous Cell Carcinoma (HNSCC) ranks as the sixth most common cancer globally and remains a clinically challenging disease due to its high rates of local recurrence and distant metastasis. Its incidence continues to rise, with projections indicating a 30% increase by 2030, corresponding to approximately 1.08 million new cases annually [1]. Advanced HNSCC often involves structures of the head and neck region, including the nasal cavity and pharynx, leading to impaired chewing and swallowing functions, facial disfigurement, and speech disorders. These deficits profoundly diminish patients’ quality of life [2]. Moreover, the five-year survival rate for HNSCC remains among the lowest of all solid malignancies and has stagnated at around 50% over the past three decades [3]. Beyond direct mortality, HNSCC survivors exhibit a notably high suicide rate of 63.4 per 100,000—the second highest among all cancer survivors, after pancreatic cancer (86.4 per 100,000), and significantly exceeding that of other cancer survivors (23.6 per 100,000). Psychological distress and reduced quality of life are considered major contributing factors [4]. The high mortality of HNSCC stems from two key challenges: (1) the lack of reliable biomarkers for early diagnosis, and (2) the absence of specific molecular targets to guide precision therapy and improve prognosis [5]. Furthermore, squamous cell carcinomas originating from different anatomical subsites within the head and neck region demonstrate distinct epidemiological characteristics and tumor progression mechanisms, reflecting the heterogeneity and complexity of the disease [6]. This variability further complicates the development of effective treatment strategies, underscoring the need for in-depth investigation into the molecular mechanisms and potential biomarkers of HNSCC.

Non-coding RNAs (ncRNAs) represent a class of RNA molecules that do not encode proteins, such as miRNAs, lncRNAs, circRNAs, and others. They play critical roles in the epigenetic, transcriptional, and post-transcriptional regulation of gene expression, and are involved in a wide range of biological processes including cell differentiation, development, metabolism, and disease pathogenesis [7,8]. In cancer, ncRNAs function as key regulators, acting either as oncogenes to promote malignant phenotypes—such as proliferation, invasion, metastasis, and drug resistance—or as tumor suppressors that inhibit cancer progression. These molecules contribute to tumorigenesis through diverse mechanisms, including miRNA-mediated mRNA degradation, lncRNA-involved epigenetic regulation, circRNA-based ceRNA networks, as well as modulation of the tumor microenvironment and immune responses [9,10]. Moreover, ncRNAs demonstrate considerable clinical potential as diagnostic and prognostic biomarkers, and as therapeutic targets in cancer [11,12].

In head and neck squamous cell carcinoma (HNSCC), lncRNAs can act as oncogenes or tumor suppressors by influencing tumor proliferation, invasion, and metastasis via mechanisms such as epigenetic modification, transcriptional regulation, and competitive binding to miRNAs. As such, they are regarded as potential diagnostic biomarkers and therapeutic targets [13]. Recent studies have identified a number of differentially expressed lncRNAs—including *PVT1*, *LINC00491*, *MALAT1*, *HOXA11-AS*, *HCG22*, and *LINC01133*—that participate in HNSCC progression through various molecular pathways [14,15,16,17,18,19].

LncRNA *NEAT1* (Nuclear Enriched Abundant Transcript 1), one of the earliest identified long non-coding RNAs, is predominantly localized in the nucleus where it plays a critical role in the formation and organization of paraspeckles. Studies have demonstrated that LncRNA *NEAT1* is commonly upregulated in a variety of solid tumors [20]. lncRNA *NEAT1* is significantly up-regulated in a wide range of cancer types, including those of the head and nervous systems (glioma, oral squamous cell carcinoma, laryngeal squamous cell carcinoma, nasopharyngeal carcinoma) [21,22,23,24], digestive system (gastric cancer, colon cancer, esophageal cancer, liver cancer, and pancreatic cancer) [25,26,27,28,29], respiratory system (lung adenocarcinoma, lung squamous cell carcinoma, non-small cell lung cancer) [30,31,32], urinary system (renal cell carcinoma, bladder cancer, prostate cancer) [33,34,35], gynecological system (cervical cancer, ovarian cancer, and endometrial cancer) [36,37,38], endocrine system (thyroid cancer, breast cancer) [39,40], as well as other systems (retinoblastoma, melanoma) [41,42].

**Figure 1:**
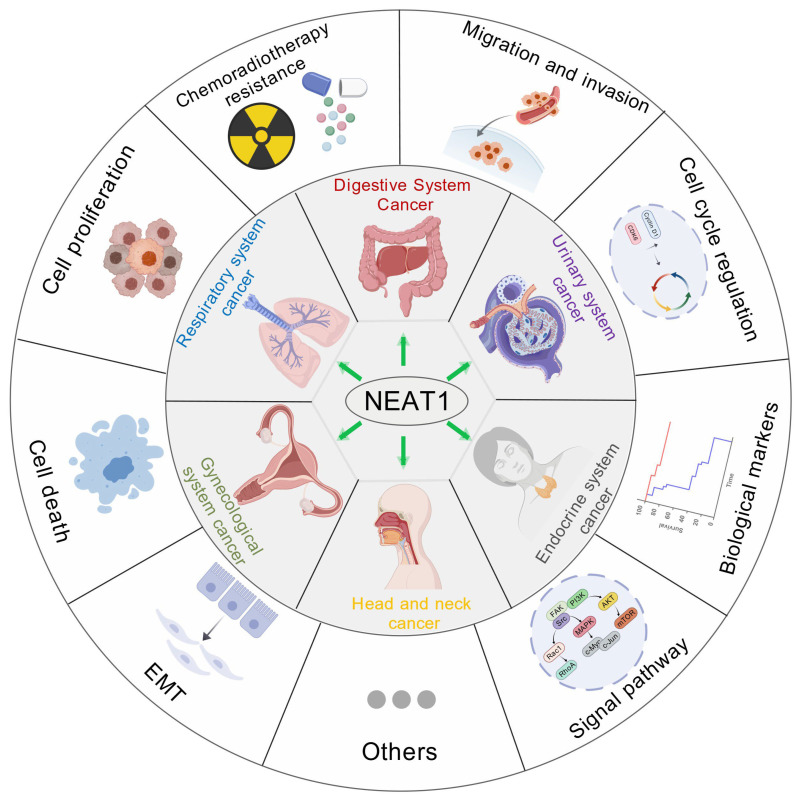
The role of lncRNA *NEAT1* in human cancers.

In these cancer types, lncRNA *NEAT1* promotes tumor development through diverse molecular mechanisms. These include its role as a miRNA sponge, where it sequesters miRNAs and prevents them from mediating the degradation of target genes, thereby regulating multiple signaling pathways—such as VEGF, Notch, and Wnt/β-catenin—to facilitate tumor cell proliferation, migration, and angiogenesis [43,44]. Additionally, lncRNA *NEAT1* participates in the regulation of apoptosis and cell cycle progression, influences cancer stem cell properties, and modulates immune cell functions within the tumor microenvironment [45,46]. Notably, under hypoxic conditions, lncRNA *NEAT1* expression is induced by HIF-1α, further driving malignant progression [47,48]. Clinically, elevated lncRNA *NEAT1* expression is closely associated with poor patient outcomes, supporting its utility as a diagnostic and prognostic biomarker in various cancers [49] (Figure 1).

In summary, the clinical management of head and neck squamous cell carcinoma faces multiple challenges, including tumor heterogeneity, therapy resistance, and a lack of precise biomarkers. A deeper understanding of the role of lncRNAs, particularly lncRNA *NEAT1*, in tumor biology is crucial for elucidating its oncogenic mechanisms and discovering novel diagnostic and therapeutic targets for HNSCC.

Future research will require the integration of multidisciplinary approaches and cutting-edge technologies. Utilizing single-cell and spatial transcriptomics will enable the precise mapping of lncRNA *NEAT1* expression across distinct cellular compartments within the tumor and its microenvironment [50]. Patient-derived organoid models will provide a physiologically relevant platform to dissect lncRNA *NEAT1*’s functional roles and test targeted interventions in a controlled yet complex setting [51]. Furthermore, applying machine learning algorithms to multi-omics data will be instrumental in deciphering the comprehensive lncRNA *NEAT1* regulatory network, predicting its crosstalk with signaling pathways and the tumor immune microenvironment, and ultimately building robust prognostic or predictive models [52]. This concerted effort aims to advance the development of precision therapy and improve patient outcomes.

## 2. The Structural Characteristics of Long Non-Coding RNA *NEAT1*

LncRNA *NEAT1* (Nuclear Enriched Abundant Transcript 1) is located on chromosome 11q13.1 and gives rise to two major isoforms with distinct structures and functional specializations: the shorter *NEAT1_1* (∼3.7 kb) and the longer *NEAT1_2* (∼23 kb) [53]. These two isoforms are generated through alternative transcription termination and processing. The shorter transcript, *NEAT1_1*, is produced via canonical polyadenylation and termination. In contrast, the *NEAT1_2* isoform is transcribed when RNA polymerase II reads through the termination signal; its 3ʹ-end, generated by RNase P cleavage, is stabilized by a triple-helix structure rather than a canonical poly(A) tail [54].

Although *NEAT1_1* is a linear transcript that does not participate in paraspeckle formation, recent studies have demonstrated that it is not functionally inert [55]. In glioblastoma, the expression ratio of *NEAT1_1* to *NEAT1_2* is dysregulated, with *NEAT1_1* being more prominently upregulated. Deletion of the poly(A) signal of lncRNA *NEAT1* using CRISPR–Cas9 significantly reduced *NEAT1_1* expression while increasing that of *NEAT1_2*. This imbalance led to enhanced assembly of nuclear paraspeckles (increased in number), promoted glioma cell migration, and widespread transcriptomic alterations—including upregulation of gene pathways associated with cell migration, adhesion, and polarity. These effects were predominantly driven by the increase in *NEAT1_2* rather than the decrease in *NEAT1_1*, and could be reversed by *NEAT1_2*-specific knockdown [56]. *NEAT1_2* possesses a unique structural feature: inverted repeats at its 3ʹ-end enable the formation of a triple-stranded RNA structure that confers self-stability, allowing it to serve as the core architectural scaffold for the nuclear subdomain known as the paraspeckle. This closed circular topology provides *NEAT1_2* with high stability, enabling it to form a regulatory hub within the nucleus—analogous to a membrane-bound organelle—by recruiting various RNA-binding proteins (e.g., SFPQ, NONO) and participating in essential processes such as transcriptional regulation, stress response, RNA editing, and genome stability maintenance [55,57,58,59].

**Figure 2:**
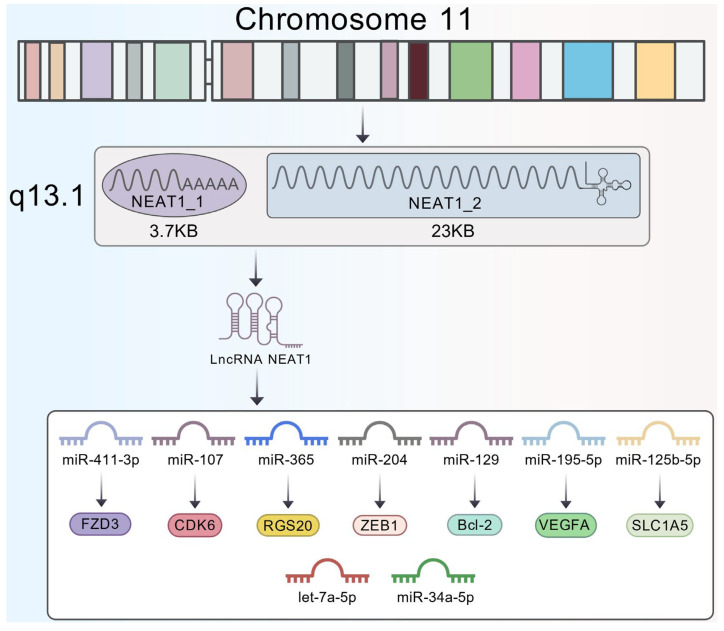
Structural Characteristics of lncRNA *NEAT1*.

LncRNA *NEAT1* plays a pivotal oncogenic role in tumor progression. It promotes cancer cell proliferation, invasion, metastasis, and chemotherapy resistance, largely through dysregulating key signaling pathways such as Wnt/β-catenin and PI3K/AKT [60,61]. In head and neck squamous cell carcinoma (HNSCC), lncRNA *NEAT1* promotes tumor progression through multiple regulatory axes—such as miR-411-3p/*FZD3* [62], miR-365/*RGS20* [22], miR-107/*CDK6* [23], and miR-204/*ZEB1* [63]—mediating diverse oncogenic mechanisms and highlighting its potential as a therapeutic target in HNSCC (Figure 2). Importantly, it should be noted that most functional studies in HNSCC to date have not distinguished between the two major isoforms (*NEAT1_1* and *NEAT1_2*). Thus, the mechanisms summarized in the following sections primarily reflect the collective functions attributed to *NEAT1* in the existing literature.

## 3. The Mechanism by Which lncRNA *NEAT1* Regulates the Progression of HNSCC

### 3.1. Regulation of Cell Proliferation and Death

The dynamic imbalance between cell proliferation and death serves as a fundamental driver of malignant transformation and cancer progression. Uncontrolled proliferation, mediated by dysregulation of signaling pathways such as Cyclin D-*CDK4/6-Rb* and PI3K/AKT, enables sustained tumor growth [64,65], while evasion of apoptosis—through mechanisms like *BCL-2* overexpression or *p53* inactivation—ensures aberrant cell survival [66,67]. This dual dysregulation not only accelerates tumorigenesis and metastasis but also underlies resistance to conventional therapies. Therapeutic approaches targeting the proliferation death axis involve two main strategies: inhibiting cell proliferation using agents such as CDK4/6 inhibitors (e.g., palbociclib) and *EGFR* inhibitors (e.g., cetuximab) [68,69], and restoring programmed cell death via *BCL-2* antagonists (e.g., venetoclax) or ferroptosis inducers (e.g., erastin) [70,71]. These processes are further modulated by the tumor microenvironment (TME), where immune cells such as T lymphocytes can induce apoptosis through the *Fas/FasL* pathway, and cancer-associated fibroblasts (CAFs) secrete growth factors like *HGF* and *IGF-1* to promote proliferation and suppress apoptosis [72,73]. A comprehensive therapeutic strategy should therefore target both tumor-intrinsic mechanisms and the dynamic crosstalk between cancer cells and their microenvironment. Simultaneously targeting both processes—for instance, combining CDK4/6 inhibitors to restore cell-cycle control with BH3 mimetics to reactivate apoptosis—represents a promising direction in precision oncology [74,75].

Studies have shown that lncRNA *NEAT1* is highly expressed across various subtypes of HNSCC, its upregulation is correlated with advanced tumor stage and lymph node metastasis, suggesting its tumor-promoting function [62]. Through the competitive endogenous RNA (ceRNA) mechanism.

**Figure 3:**
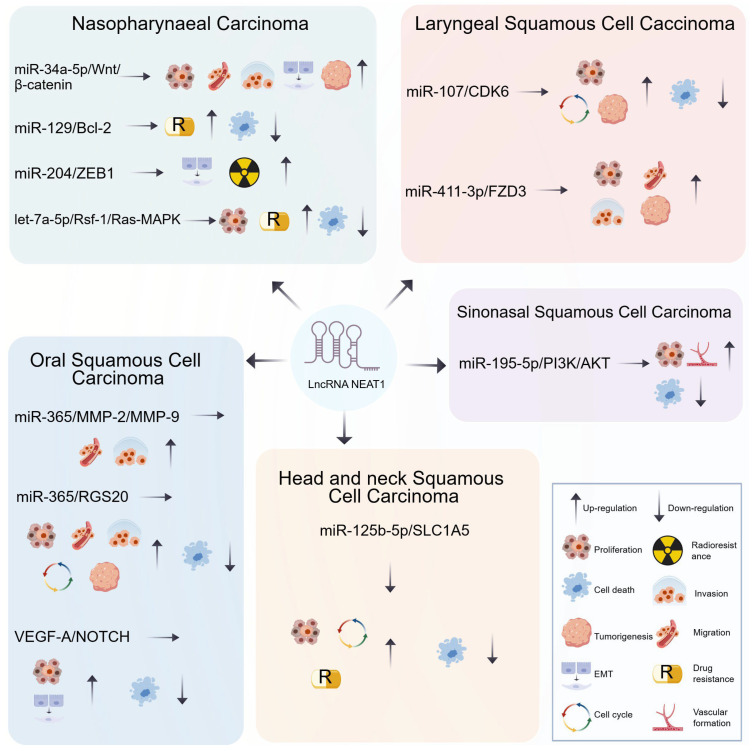
The mechanism by which lncRNA *NEAT1* regulates the malignant phenotypes of various subtypes of HNSCC.

lncRNA *NEAT1* facilitates cell growth by sequestering multiple microRNAs and dysregulating their downstream targets, These include miR-34a-5p (via the WNT/β-catenin axis) [76], let-7a-5p (via the RASSF1/Ras-MAPK axis), miR-411-3p (via *FZD3*) [62], miR-107 (via *CDK6*) [23], miR-365 (via *RGS20*) [22,77], miR-195-5p (via *VEGFA*) [78], and miR-125b-5p (via *SLC1A5*) [79]. Moreover, lncRNA *NEAT1* enhances cell survival and chemoresistance by regulating the miR-125b-5p/*SLC1A5* axis, which promotes glutamate transport and metabolic reprogramming [79]. Downregulation of lncRNA *NEAT1* has been shown to increase oxidative stress, promote apoptosis and autophagy, and thereby suppress tumor proliferation [79]. Regarding the suppression of cell death, lncRNA *NEAT1* also inhibits cell death programs by targeting several regulatory axes, such as miR-195-5p (via *VEGFA*) [78], let-7a-5p (via the *RASSF1*/Ras-MAPK axis) [24], miR-107 (via *CDK6*) [23], miR-125b-5p (via *SLC1A5*) [79], miR-365 (via *RGS20*) [75], and miR-129 (via *Bcl-2*) [80]. Notably, its sequestration of miR-195-5p also promotes angiogenesis, thereby providing nutritional support for tumor progression [78]. Furthermore, high lncRNA *NEAT1* expression is associated with altered immune cell infiltration patterns in tumor tissues, suggesting its role in modulating immune evasion within the TME, which may indirectly promote HNSCC cell survival and proliferation [79] (Figure 3).

Through its function as a competing endogenous RNA (ceRNA), lncRNA *NEAT1* forms a finely-tuned core network that critically regulates the “proliferation-death” balance in HNSCC cells. However, it remains unclear whether hierarchical or synergistic relationships exist among these distinct miRNA axes, and how lncRNA *NEAT1* interacts with specific cell types within the tumor microenvironment to indirectly influence this equilibrium. Future investigations should validate these mechanisms in more physiologically relevant models, such as organoid co-culture systems, and explore whether targeting lncRNA *NEAT1* can selectively reverse the dysregulation of proliferation and apoptosis in cancer cells while minimizing toxicity to normal tissues [81].

### 3.2. The Role in Epithelial–Mesenchymal Transition

Epithelial–mesenchymal transition (EMT) plays a pivotal role in the development and progression of head and neck squamous cell carcinoma (HNSCC). EMT is a biological process in which epithelial cells lose their characteristic features and acquire mesenchymal traits, a transition closely associated with enhanced invasiveness and metastatic potential of tumor cells [82,83]. Studies have demonstrated that in HNSCC, EMT not only facilitates tumor cell migration and invasion but also correlates with poor clinical outcomes, including lymph node metastasis and higher pathological grades [84,85].

In-depth analysis of the role of EMT in HNSCC reveals its underlying mechanisms and clinical implications. On one hand, EMT promotes the transition of tumor cells to a mesenchymal phenotype by regulating multiple signaling pathways, such as the transforming growth factor-β (TGF-β) pathway, thereby enhancing their migratory and invasive capabilities [1,86]. Moreover, the morphological and molecular changes during EMT may impair immune recognition and attack of tumor cells, further facilitating immune escape [87]. On the other hand, key factors such as *TWIST1* and *IGF2BP1* have been found to regulate the EMT process, and their expression levels are closely associated with patient prognosis, providing new targets for individualized therapy [88,89].

Among them, lncRNA *NEAT1* plays a key role in HNSCC: it promotes EMT by modulating the *VEGF-A*/Notch axis, characterized by down-regulation of *E-cadherin* and up-regulation of *N-cadherin*, *vimentin*, and *Snail* [90]. Additionally, lncRNA *NEAT1* can sequester microRNAs such as miR-34a-5p (via WNT/β-catenin) and miR-204 (via *ZEB1*), thereby relieving their repression on target genes or directly enhancing the EMT program [63,76], ultimately influencing tumor cell metastatic and invasive processes (Figure 3).

Accumulating evidence supports the consensus that lncRNA *NEAT1* acts as a pleiotropic regulator driving the EMT program in HNSCC. It functions by sequestering multiple EMT-suppressive miRNAs, thereby relieving their repression on key EMT-related transcription factors including *ZEB1* and *Snail*. Nevertheless, several important questions remain. For instance, are these distinct lncRNA *NEAT1*/miRNA axes universally activated across all HNSCC subtypes, or are they selectively engaged under specific genetic backgrounds or microenvironmental cues? Furthermore, whether lncRNA *NEAT1*-mediated EMT is associated with full reversibility (i.e., mesenchymal-to-epithelial transition) and what role it plays in maintaining cancer stem-like properties require further clarification.

### 3.3. Regulation of Invasion and Migration

During the progression of head and neck squamous cell carcinoma (HNSCC), the migratory and invasive capabilities of tumor cells represent critical determinants of malignancy. Accumulating evidence indicates that tumor cells employ diverse mechanisms to enhance their mobility and invasiveness, ultimately leading to more severe clinical outcomes.

Long non-coding RNAs (lncRNAs) have emerged as important regulatory factors in modulating tumor cell migration and invasion. These molecules influence gene expression through multiple mechanisms, such as functioning as miRNA sponges or directly regulating target genes, thereby actively participating in HNSCC pathogenesis [91]. Furthermore, lncRNAs may enhance the migratory capacity of tumor cells by modulating the epithelial–mesenchymal transition (EMT) process, which is critical for the invasive behavior of HNSCC [92,93]. Current studies indicate that lncRNA *NEAT1* is upregulated in metastatic HNSCC cells compared to primary cancer cells [94]. Furthermore, multiple reports have detected higher expression levels of lncRNA *NEAT1* in tissues from cervical lymph node metastases and advanced-stage HNSCC, a finding further supported by meta-analyses [23,76,77].

Across various subtypes of head and neck squamous cell carcinoma (HNSCC), lncRNA *NEAT1* enhances cellular migration and/or invasion through multiple miRNA/target gene signaling axes. These include the miR-34a-5p/WNT/β-catenin axis [76], the miR-125b-5p/*SLC1A5* axis [79], the miR-411-3p/*FZD3* axis [62], the miR-365/*RGS20* axis [22], and the miR-365/*MMP-2*/*MMP-9* axis [77]. These mechanisms collectively underscore the multi-pathway synergy of lncRNA *NEAT1* in regulating the invasive and metastatic processes of HNSCC (Figure 3).

In summary, lncRNA *NEAT1* drives the migration and invasion of HNSCC cells through an overlapping and redundant ceRNA network involving miRNAs. This constitutes a robust and well-supported consensus mechanism. The functional convergence of these axes may reflect the network robustness evolved by tumor cells to sustain metastatic competence. However, how these mechanisms are precisely sequenced or coordinated during the spatiotemporal dynamics of invasion and metastasis—such as from local invasion to intravasation, circulatory survival, extravasation, and colonization—remains to be elucidated. Furthermore, whether lncRNA *NEAT1* influences migration by more directly regulating physical processes, such as cytoskeletal dynamics or cell–matrix adhesion, awaits further experimental investigation.

### 3.4. Regulation of Signaling Pathways

The regulation of signaling pathways is critically important in tumor initiation and progression. Dysregulation of several classic signaling cascades—such as PI3K/AKT, RAS/MAPK, Wnt/β-catenin, and Notch—constitutes a central mechanism driving malignant transformation in cancer cells. Abnormal activation or inhibition of these pathways collectively promotes tumor progression and metastasis by modulating key cellular processes, including proliferation, apoptosis, metabolism, invasion, migration, angiogenesis, and tumor microenvironment remodeling [95,96,97,98,99]. Therefore, elucidating the regulatory networks and interactions of specific signaling pathways in tumors not only aids in uncovering the mechanisms of tumor pathogenesis but also facilitates the development of novel molecularly targeted therapeutic strategies.

**Figure 4:**
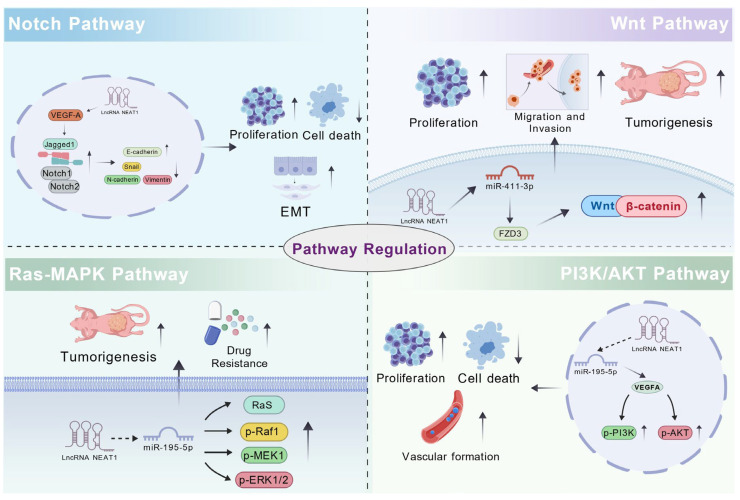
The mechanism by which lncRNA *NEAT1* regulates the signaling pathways in HNSCC.

Specifically, lncRNA *NEAT1* contributes to HNSCC progression by orchestrating the activation of multiple oncogenic signaling pathways. It activates the Wnt/β-catenin pathway through the miR-411-3p/*FZD3* axis [62]. In parallel, it promotes PI3K/AKT signaling via the miR-195-5p/*VEGFA* axis, indicated by increased p-PI3K and p-AKT levels [78]. Additionally, lncRNA *NEAT1* upregulates *VEGF-A* to stimulate the Notch pathway [90], while also activating the Ras-MAPK cascade through the let-7a-5p/*Rsf-1* axis, evidenced by elevated expression of Ras, p-Raf1, p-MEK1, and p-ERK1/2 [24]. Together, these coordinated mechanisms mediated by lncRNA *NEAT1* drive the malignant phenotype and advance tumor progression in HNSCC (Figure 4).

Collectively, lncRNA *NEAT1* functions not as a regulator of a single pathway but as a signaling “cross-hub.” Through its ceRNA activity, it concurrently modulates multiple core oncogenic pathways. This multi-pathway regulatory capacity likely underlies the pleiotropic pro-tumorigenic functions of lncRNA *NEAT1*. Nevertheless, several important questions remain: whether substantial cross-talk exists among these lncRNA *NEAT1*-activated pathways; whether lncRNA *NEAT1* itself is subject to feedback regulation by downstream effectors of these pathways; and, in different HNSCC subtypes or microenvironmental contexts, which pathway serves as the predominant effector of lncRNA *NEAT1*-driven oncogenesis.

### 3.5. Diagnostic and Prognostic Biomarkers

Despite accumulating evidence suggesting the potential of long non-coding RNA *NEAT1* (lncRNA *NEAT1*) as a diagnostic and prognostic biomarker in various common malignancies, a systematic summary of its role in head and neck squamous cell carcinoma (HNSCC) remains lacking. For instance, Meta-analyses have indicated that elevated lncRNA *NEAT1* expression is associated with poor prognosis in patients with multiple cancer types and may serve as a predictor of clinicopathological features [100]. Furthermore, Shieh et al. demonstrated that high lncRNA *NEAT1* expression correlates with unfavorable survival outcomes in HNSCC patients, indicating poorer prognosis even after radiotherapy and chemotherapy [101]. Similarly, Yuan et al., using Mendelian randomization and eQTL analysis, revealed that the lncRNA *NEAT1* rs3741384 GG genotype predicts adverse prognosis in HNSCC patients, suggesting that genetic variations in lncRNA *NEAT1* may influence patient survival [102].

In terms of diagnostic value, lncRNA *NEAT1* has also been recognized as a diagnostic biomarker in several human cancers [103]. In HNSCC, tumor tissues exhibit significantly higher levels of lncRNA *NEAT1* compared to adjacent non-tumor tissues (3.041 ± 0.709-fold, *p* < 0.01). LncRNA *NEAT1* expression is significantly correlated with tumor T stage, cervical lymph node metastasis, and clinical stage in HNSCC, with higher expression observed in T3–T4 tumors, lymph node metastases, distant metastases, and advanced clinical stages [23,104,105].

Notably, multiple studies in HNSCC have consistently reported that upregulated lncRNA *NEAT1* expression is strongly associated with adverse clinical outcomes (Table 1), further supporting its reliability as a prognostic indicator and potential therapeutic target. Moreover, the diagnostic utility of lncRNA *NEAT1* in HNSCC has been confirmed by several investigations (Table 1), highlighting its considerable potential as a clinical diagnostic biomarker.

A clear consensus has emerged from current research: lncRNA *NEAT1* is highly expressed in HNSCC tissues, and its expression level shows a significant positive correlation with advanced tumor stage, lymph node metastasis, and poor prognosis. These findings strongly support its potential as a promising diagnostic and prognostic biomarker. However, most evidence is derived from tissue samples; therefore, its stability and diagnostic performance in liquid biopsy sources such as plasma or saliva require validation in prospective, large-scale cohorts [107]. Another major challenge lies in establishing a universally applicable and standardized detection threshold (cut-off value). Finally, integrating lncRNA *NEAT1* with other established markers (e.g., HPV status, PD-L1) or emerging non-coding RNAs into a multi-molecular signature may offer greater predictive value than a single biomarker alone, representing a critical direction for future research [108].

### 3.6. Resistance to Therapeutic Agents and Strategies Targeting lncRNA NEAT1

Chemoresistance represents a major cause of treatment failure and poor prognosis in patients with head and neck squamous cell carcinoma (HNSCC) [109]. Cisplatin-based chemotherapy regimens have become a cornerstone of systemic treatment for HNSCC [110]. However, the intrinsic or acquired resistance of tumor cells significantly limits its clinical efficacy. Therefore, elucidating the underlying molecular mechanisms of chemoresistance in HNSCC and identifying potential targets to reverse drug resistance hold considerable scientific and clinical value for improving patient outcomes [111].

**Figure 5:**
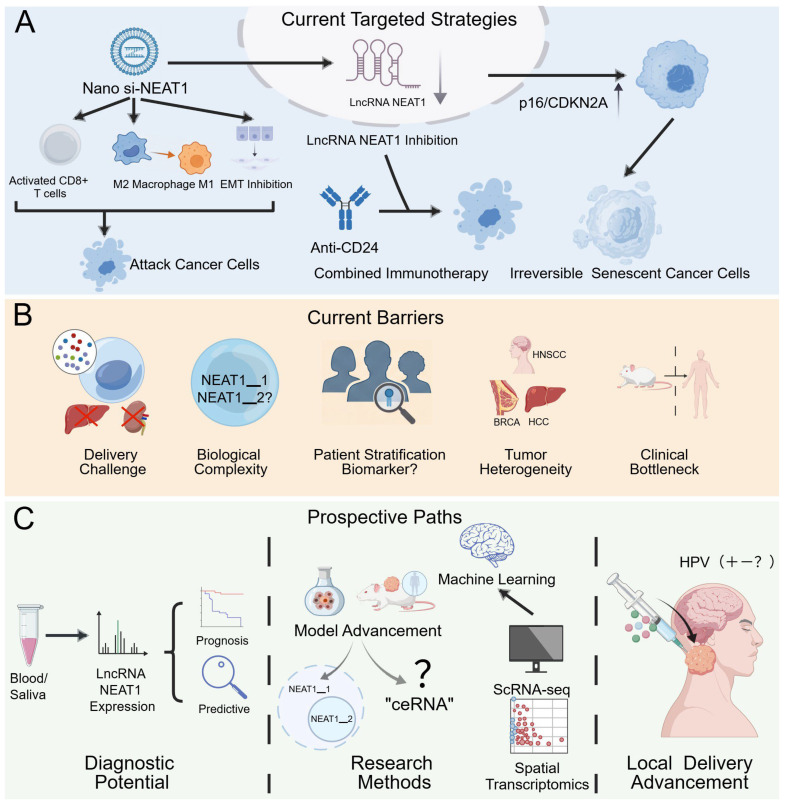
(**A**) Current therapeutic strategies targeting LncRNA *NEAT1* in cancer. (**B**) Obstacles to clinical translation. (**C**) Future directions: potential applications and research priorities for lncRNA *NEAT1*. (In this figure, “+”, “−”, and “?” indicate positive, negative, and undetermined status, respectively).

In recent years, the role of non-coding RNAs (ncRNAs) in tumorigenesis, progression, and therapy resistance has garnered increasing attention. Through involvement in complex regulatory networks, ncRNAs influence multiple biological processes—such as cell proliferation, apoptosis, epithelial–mesenchymal transition (EMT), and tumor microenvironment remodeling—thereby modulating drug resistance in cancers [7,112,113]. Multiple studies have revealed that lncRNA *NEAT1* is significantly upregulated in HNSCC and closely associated with adverse pathological features and poor prognosis, suggesting its role as a key molecule promoting malignant progression. Notably, lncRNA *NEAT1* plays an important role in regulating therapeutic sensitivity in HNSCC. For example, high expression of lncRNA *NEAT1* significantly reduces the survival rate of HNSCC patients, even after radiotherapy or chemotherapy [101]. Furthermore, existing studies indicate that lncRNA *NEAT1* promotes migration, invasion, and cisplatin resistance in HNSCC cells via the miR-125b-5p/*SLC1A5* axis [79]; influences cisplatin resistance and tumorigenicity in nude mice through regulation of let-7a-5p [24]; and modulates EMT and radioresistance in HNSCC via the miR-204/*ZEB1* axis [63] (Figure 5A).

These findings suggest that lncRNA *NEAT1* may exert pleiotropic regulatory functions across different drug treatment contexts. LncRNA *NEAT1* shows promise not only as a biomarker for predicting therapeutic response but also as a potential target for enhancing the efficacy of existing regimens and developing novel intervention strategies.

Targeting lncRNA *NEAT1* has emerged as a promising novel direction in cancer therapeutics. LncRNA *NEAT1* is frequently overexpressed in multiple cancer types and promotes tumor progression through diverse mechanisms, including participation in DNA repair, modulation of the immune microenvironment, and regulation of cellular senescence. Current targeting strategies primarily involve the following four approaches: One key strategy utilizes nanoparticle-based delivery of siRNA or shRNA to specifically silence lncRNA *NEAT1*. For example, Chen et al. employed layered double hydroxide (LDH) nanoparticles to deliver si-NEAT1 (LDH@si-NEAT1), which activated CD3+CD8+ T cells, inhibited immune evasion in breast cancer cells, polarized M2-type TAMs towards an M1 phenotype, suppressed EMT, and remodeled the immunosuppressive microenvironment in mouse models, thereby synergizing with anti-PD-1 therapy to enhance antitumor immunity [114]. Additionally, combining lncRNA *NEAT1* inhibition with existing immune checkpoint inhibitors—such as anti-CD24 antibodies—has been shown to significantly suppress tumor growth and counteract immune escape [115]. Another intriguing study in liver cancer demonstrated that inhibiting lncRNA *NEAT1* relieves its repression of senescence-associated pathways (e.g., *CDKN2A*/p16), driving cancer cells into irreversible senescence. This finding provides a novel rationale for exploiting “therapy-induced senescence” as a potential treatment strategy [116] (Figure 5A).

However, it is important to note that all the findings summarized above remain at the preclinical stage, and most are not based on HNSCC-specific models. LncRNA *NEAT1*-targeted therapy has not yet entered clinical trials for any cancer, and its translation faces multiple practical hurdles: (1) Delivery and specificity challenges: lncRNA *NEAT1* is predominantly localized in the nucleus, necessitating efficient, specific, and safe delivery of siRNA/ASOs into the nuclear compartment—a key technical hurdle for RNA interference. Current nanodelivery systems still require optimization in terms of targeting efficiency, organ selectivity, and immunogenicity. Off-target effects could lead to unpredictable toxicity in normal tissues [117]. (2) Biological complexity of the target: lncRNA *NEAT1*, particularly *NEAT1_2*, serves as the functional core of nuclear paraspeckles, which are involved in normal stress responses, RNA processing, and the maintenance of genome stability. Global inhibition of lncRNA *NEAT1* may disrupt these fundamental physiological processes, posing long-term safety risks [118]. (3) Unclear patient stratification and beneficiary population: HNSCC exhibits high heterogeneity, and it remains unclear which subtypes or molecular subgroups would benefit most from lncRNA *NEAT1* inhibition. The lack of reliable predictive biomarkers significantly increases the risk of clinical trial failure [119]. (4) Tumor heterogeneity: The function and regulatory mechanisms of lncRNA *NEAT1* may vary across cancer types and even among different subtypes of the same cancer, necessitating further clarification of its context-dependent roles [120]. (5) Clinical translation bottleneck: All significant progress remains confined to preclinical studies, and rigorous human trials are required to validate both safety and efficacy (Figure 5B) [121,122].

Given these fundamental challenges, advancing lncRNA *NEAT1* as a predictive biomarker may currently represent a more translationally feasible path than its direct development as a therapeutic target. Its high expression is closely associated with poor prognosis and treatment resistance, supporting its role as a prognostic indicator [101,106]. Further prospective studies could validate whether lncRNA *NEAT1* expression levels—detected via liquid biopsies such as blood or saliva—can predict patient responses to specific radiotherapy or chemotherapy regimens, thereby guiding pre-treatment risk stratification and personalized therapeutic strategies. This approach entails significantly lower technical and regulatory barriers compared with developing novel RNA-targeting drugs [123] (Figure 5C).

In summary, although the role of lncRNA *NEAT1* in mediating therapy resistance in HNSCC is increasingly clear, its direct targeting as a therapeutic agent remains a formidable challenge. Future research should proceed along two parallel tracks: first, rigorously evaluating the efficacy and safety of lncRNA *NEAT1* inhibition in more clinically relevant HNSCC models (e.g., patient-derived organoids) and exploring strategies such as localized delivery to reduce systemic toxicity; second, prioritizing the multicenter validation of lncRNA *NEAT1* as a clinical biomarker—a pathway that may currently offer a faster and more practical route toward clinical translation.

## 4. Conclusions

This review systematically elucidates the pleiotropic oncogenic functions of the long non-coding RNA *NEAT1* (LncRNA *NEAT1*) in head and neck squamous cell carcinoma (HNSCC), establishing its central role in regulating critical processes such as tumor proliferation, metastasis, and therapy resistance via a complex ceRNA network (Figure 3). However, significant limitations and unresolved questions persist in the current body of research, delineating key directions for future investigation.

First, the function of lncRNA *NEAT1* exhibits substantial context-dependency. HNSCC itself is highly heterogeneous in terms of anatomical origin, etiology (e.g., HPV infection status), and tumor microenvironment [124]. Although lncRNA *NEAT1* displays pro-tumorigenic properties across multiple subtypes, its upstream regulatory signals, predominant downstream pathways, and clinical performance as a biomarker likely vary. For instance, A key regulatory axis involves the tumor suppressor p53, which directly binds to the *NEAT1* promoter and activates its transcription. This p53-mediated induction of *NEAT1* contributes to the tumor-suppressive function of p53, forming a reciprocal transcriptional regulatory network [125]. Consequently, any upstream event affecting p53 stability or function may perturb *NEAT1* expression. In HPV-positive HNSCC, the viral oncoprotein E6 binds to the host ubiquitin ligase E6AP, forming a complex that specifically targets p53 for ubiquitin-proteasome-mediated degradation. This constitutes a core mechanism of HPV-driven carcinogenesis, leading directly to the loss of p53 tumor-suppressor activity [126]. From this, the following regulatory cascade can be deduced: HPV positivity → E6 overexpression → p53 degradation → impaired transcriptional activity of p53 → potential suppression or dysregulation of its downstream target gene *NEAT1*. Thus, in HPV-positive cases, E6-mediated p53 degradation likely reduces p53 transcriptional activity, thereby weakening its induction of *NEAT1* and potentially leading to suppressed or aberrant *NEAT1* expression. In contrast, in HPV-negative HNSCC—particularly those associated with smoking—*TP53* frequently acquires inactivating or gain-of-function mutations due to tobacco exposure [127]. These mutations result in loss of normal p53 transcriptional activity or acquisition of oncogenic functions, which can also drive *NEAT1* dysregulation. The patterns of *NEAT1* dysregulation and the associated biological contexts (e.g., genomic instability, oxidative stress) thus differ fundamentally between HPV-positive and HPV-negative tumors. Notably, the biological role of *NEAT1* in tumors is dynamically determined by p53 status: it exerts a tumor-suppressive role in synergy with functional p53, whereas upon p53 inactivation, *NEAT1* may shift toward promoting tumorigenesis [128]. This context-dependent mechanism holds significant implications for both HPV-positive and HPV-negative head and neck squamous cell carcinomas.

Beyond its context-dependent regulation by p53, *NEAT1* functions across various solid tumors as a pivotal molecular scaffold, orchestrating oncogenic gene networks by recruiting specific transcriptional or chromatin-modifying complexes. It operates through distinct modes: as a transcriptional co-regulatory platform—for example, in prostate cancer, where it directly binds transcription factor *CDC5L* to co-activate the oncogene *AGRN* [129]—and as an epigenetic regulatory hub, as seen in gastric cancer, where it mediates *ALKBH5/EZH2*-driven silencing of tumor suppressors [130]. Its own expression is also tightly controlled, such as by *MUC1*-C via PBAF-dependent chromatin remodeling [131]. This evidence underscores *NEAT1*’s role as a dynamic signal-integrating node, suggesting that in HNSCC, it may similarly recruit HPV-status-specific protein complexes to drive divergent downstream pathways, thereby contributing to the distinct phenotypes observed in HPV-positive and HPV-negative tumors. Currently, the specific mechanisms by which *NEAT1* recruits transcriptional or chromatin-modifying complexes in HNSCC remain insufficiently explored. Therefore, future research should systematically map the *NEAT1*-interacting proteome in HNSCC to decode its precise, context-dependent regulatory circuits. It is also imperative to validate the association between *NEAT1* expression and prognosis or therapeutic response in well-stratified clinical cohorts and corresponding experimental models (e.g., patient-derived organoids), based strictly on anatomical subsites and HPV status [119,132].

Second, the functional complexity of lncRNA *NEAT1* extends far beyond our current understanding. On one hand, its two major transcript isoforms, *NEAT1_1* and *NEAT1_2*, likely have distinct roles based on their subcellular localization, molecular interactions, and functions [55]. The long isoform *NEAT1_2* is essential for the scaffold and formation of paraspeckles, a type of nuclear body involved in various regulatory processes, while the short isoform *NEAT1_1* may exert functions independent of this structure [131]. Critically, current research in HNSCC largely overlooks the specific contributions of paraspeckles themselves to cancer progression. Whether the oncogenic functions attributed to lncRNA *NEAT1* are predominantly mediated through paraspeckle-dependent mechanisms (e.g., sequestering proteins or RNAs within the nuclear body) or through paraspeckle-independent activities of individual isoforms remains poorly defined [131]. This gap underscores a significant limitation in the field. Most existing studies treat lncRNA *NEAT1* as a homogeneous entity, obscuring which specific biological effects are mediated by which isoform. Clarifying the individual contributions and dynamic balance between *NEAT1_1* and *NEAT1_2* in HNSCC is pivotal for a deeper understanding of its molecular mechanisms. Therefore, future studies should prioritize experimental approaches that can distinguish between the two isoforms—such as isoform-specific knockdown or overexpression—to delineate their individual functions and potential synergy. Concurrently, methodologies that specifically disrupt paraspeckle architecture without abolishing lncRNA *NEAT1* expression are needed to clarify the role of this nuclear sub-structure. Together, these lines of investigation will be essential for elucidating the precise molecular mechanisms of lncRNA *NEAT1* in HNSCC and for informing the development of targeted therapeutic strategies, whether they are isoform-specific or pathway-specific.

On the other hand, the role of lncRNA *NEAT1* in shaping the HNSCC immune microenvironment remains largely unexplored. Although studies in other cancers suggest lncRNA *NEAT1* can induce CD8^+^ T cell exhaustion via axes such as miR-155/Tim-3 [133], it is still unknown whether and how lncRNA *NEAT1* differentially regulates immune cell infiltration and checkpoint expression in the immunologically distinct contexts of HPV-positive versus HPV-negative HNSCC. Systematic investigation using models like co-culture organoids or humanized mice is warranted [134] (Figure 5C).

Similarly, numerous other lncRNAs, such as lncRNA *PVT1* and lncRNA *MALAT1* [14], have also been widely demonstrated to be involved in HNSCC progression. However, there are both similarities and differences in their roles. For instance, in terms of clinical significance, both lncRNA *NEAT1* and lncRNA *MALAT1* show potential as liquid biopsy biomarkers for early diagnosis, prognosis assessment, and treatment response prediction in HNSCC. Nevertheless, differences exist in their clinical correlations. For example, in predicting radiotherapy-related toxicities, low expression of both lncRNA *NEAT1* and lncRNA *MALAT1* is significantly associated with risks of anemia, hepatic dysfunction, and malnutrition. Yet their sensitivity and specificity in identifying specific toxicity types are not identical, suggesting that they may participate in the development of treatment-related adverse reactions through distinct molecular mechanisms [135]. This comparative perspective underscores the need to precisely define the unique and shared pathways governed by different oncogenic lncRNAs, including lncRNA *NEAT1*, within the HNSCC landscape.

Furthermore, the predominant interpretation of lncRNA *NEAT1*′s core mechanism—the ceRNA model—requires critical scrutiny. As a predominantly nuclear-localized lncRNA (especially *NEAT1_2*), the spatial logic of lncRNA *NEAT1* efficiently acting as a cytoplasmic “molecular sponge” for miRNAs under physiological conditions presents a challenge [136]. Current evidence largely relies on correlative analyses and functional rescue experiments, lacking direct, quantitative validation of the stoichiometry of its interactions with miRNAs. Future studies should employ more rigorous techniques, such as crosslinking immunoprecipitation sequencing (CLIP-seq) and single-molecule imaging, to validate or refine this prevailing model [136]. We should also explore the possibility of lncRNA *NEAT1* functioning through non-ceRNA mechanisms, such as transcriptional regulation or sequestration of nuclear factors (Figure 5C).

Finally, translating lncRNA *NEAT1* research from the bench to the clinic necessitates the integration of novel methodologies. Single-cell and spatial transcriptomics can precisely resolve lncRNA *NEAT1* expression patterns across different intratumoral cell subpopulations (e.g., cancer cells, immune cells, fibroblasts) and their impact on cellular states [137,138]. Multi-omics integrative analyses can systematically uncover the upstream genetic and epigenetic drivers of aberrant lncRNA *NEAT1* expression and reconstruct its global regulatory network [139,140]. Engineered models based on patient-derived organoids provide an ideal platform for manipulating lncRNA *NEAT1* expression within a three-dimensional, near-physiological context to observe its effects on tumor growth, immune interactions, and therapeutic response [141]. Furthermore, machine learning algorithms can mine high-throughput data to build prognostic or predictive models based on lncRNA *NEAT1* and its associated features, thereby identifying patient subgroups most likely to benefit from targeted intervention [142,143] (Figure 5C).

In conclusion, future research on lncRNA *NEAT1* should move beyond the traditional view of it as a single, homogeneous molecule and instead focus on its heterogeneity and multidimensional complexity. By leveraging cutting-edge technologies to dissect its functional mechanisms within finely stratified contexts, there is significant potential to transform lncRNA *NEAT1* from a correlative marker into a diagnostic tool and precision therapeutic target with clear clinical utility.

## Figures and Tables

**Table 1:** LncRNA *NEAT1* as prognostic and diagnostic biomarkers of HNSCC.

Number of Patients/Controls	Clinical Stage (*p*-Value)	Diagnostic Properties (AUC, *p*-Value)	Prognostic Properties (*p*-Value)	Sample Source	References
45/45			HR > 1, *p* < 0.01	Tumor tissue	Shieh et al. [106]
17/17		AUC = 0.7647, *p* < 0.01	HR > 1, *p* < 0.01	Tumor tissue	Lin et al. [101]
26/32	III-IV/I-II > 1, *p* < 0.01		HR > 1, *p* < 0.01	Tumor tissue	Zheng et al. [77]
12/11			HR > 1, *p* < 0.05	Tumor tissue	Wei et al. [22]
19/14			HR > 1, *p* < 0.05	Tumor tissue	Cui et al. [76]
48/48	III-IV/I-II > 1, *p* < 0.001		HR > 1, *p* < 0.001	Tumor tissue	Wu et al. [104]
51/51	III-IV/I-II > 1, *p* < 0.01		HR > 1, *p* < 0.05	Tumor tissue	Liu et al. [105]

## Data Availability

All figures in this review are original and created by the authors. All supporting citations are provided in the reference list.

## Abbreviations

HNSCC	Head and neck squamous cell carcinoma
lncRNAs	long non-coding RNAs
ncRNAs	non-coding RNAs
NEAT1	Nuclear Enriched Abundant Transcript 1
PAS	polyadenylation signal
TME	tumor microenvironment
CAFs	cancer-associated fibroblasts
EMT	epithelial–mesenchymal transition
TGF-β	transforming growth factor-β
ceRNA	competing endogenous RNA
eQTL	expression Quantitative Trait Loci
LDH	layered double hydroxide
miRNAs	microRNAs
circRNAs	circular RNAs
HPV	Human Papillomavirus
shRNA	short hairpin RNA
siRNA	small interfering RNA
ASOs	antisense oligonucleotides
CLIP-seq	crosslinking immunoprecipitation sequencing

## References

[1] Chen X., Zhang S., Liu C., Li G., Lu S., Wang Y., Zhang X., Huang D., Qiu Y., Liu Y. (2020). UBE2O Promotes Progression and Epithelial-Mesenchymal Transition in Head and Neck Squamous Cell Carcinoma. OncoTargets Ther..

[2] Warnakulasuriya S. (2009). Global epidemiology of oral and oropharyngeal cancer. Oral Oncol..

[3] Ren Z.H., Hu C.Y., He H.R., Li Y.J., Lyu J. (2020). Global and regional burdens of oral cancer from 1990 to 2017: Results from the global burden of disease study. Cancer Commun..

[4] Osazuwa-Peters N., Simpson M.C., Zhao L., Boakye E.A., Olomukoro S.I., Deshields T., Loux T.M., Varvares M.A., Schootman M. (2018). Suicide risk among cancer survivors: Head and neck versus other cancers. Cancer.

[5] Yu W., He X., Zhang C., Huangfu H. (2023). Transcriptomics data mining to uncover signature genes in head and neck squamous cell carcinoma: A bioinformatics analysis and RNA-sequencing based validation. Am. J. Cancer Res..

[6] Ferreira C.C. (2023). The relation between human papillomavirus (HPV) and oropharyngeal cancer: A review. PeerJ.

[7] Nemeth K., Bayraktar R., Ferracin M., Calin G.A. (2024). Non-coding RNAs in disease: From mechanisms to therapeutics. Nat. Rev. Genet..

[8] Liu X., Haugh W., Zhang Z., Huang J. (2025). Emerging Role of Long, Non-Coding RNA Nuclear-Enriched Abundant Transcript 1 in Stress- and Immune-Related Diseases. Int. J. Mol. Sci..

[9] Sempere L.F., Powell K., Rana J., Brock A.A., Schmittgen T.D. (2021). Role of non-coding RNAs in tumor progression and metastasis in pancreatic cancer. Cancer Metastasis Rev..

[10] Hotnog C.M., Bostan M., Anghelescu M., Roman V., Bleotu C., Hainarosie R., Voiosu C., Marineata S., Bostan I.S., Diaconu C.C. (2025). Long Non-Coding RNAs: Significant Drivers of Carcinogenesis Mechanisms in Head and Neck Squamous Cell Carcinoma. Curr. Issues Mol. Biol..

[11] Zhang Z., Mao C., Wu Y., Wang Y., Cong H. (2025). Application of non-coding RNAs in tumors (Review). Mol. Med. Rep..

[12] Andrade R., Ribeiro I.P., Carreira I.M., Tralhão J.G. (2024). The Diagnostic and Prognostic Potentials of Non-Coding RNA in Cholangiocarcinoma. Int. J. Mol. Sci..

[13] Majka M. (2025). Non-coding RNAs as key player in cancer diagnosis and treatment. FEBS Open Bio..

[14] Qin Z., Zhang W., Liu S., Wang Y., Peng X., Jia L. (2023). PVT1 inhibition stimulates anti-tumor immunity, prevents metastasis, and depletes cancer stem cells in squamous cell carcinoma. Cell Death Dis..

[15] Cui D., Li Z., Wei C., Zhang Q., Xiao C. (2024). Long non-coding RNA LINC00491 accelerates head and neck squamous cell carcinoma progression through regulating miR-508-3p/SATB1 axis and activating Wnt signaling pathway. Cytokine.

[16] Duan Y., Yue K., Ye B., Chen P., Zhang J., He Q., Wu Y., Lai Q., Li H., Wu Y. (2023). LncRNA MALAT1 promotes growth and metastasis of head and neck squamous cell carcinoma by repressing VHL through a non-canonical function of EZH2. Cell Death Dis..

[17] Niu X., Yang B., Liu F., Fang Q. (2020). LncRNA HOXA11-AS promotes OSCC progression by sponging miR-98-5p to upregulate YBX2 expression. Biomed. Pharmacother..

[18] Wang M., Feng Z., Li X., Sun S., Lu L. (2021). Assessment of multiple pathways involved in the inhibitory effect of HCG22 on oral squamous cell carcinoma progression. Mol. Cell. Biochem..

[19] Li H., Yang Z., Yang X., Zhang F., Wang J., Wu Z., Wanyan C., Meng Q., Gao W., Yang X. (2022). LINC01123 promotes immune escape by sponging miR-214-3p to regulate B7-H3 in head and neck squamous-cell carcinoma. Cell Death Dis..

[20] Clemson C.M., Hutchinson J.N., Sara S.A., Ensminger A.W., Fox A.H., Chess A., Lawrence J.B. (2009). An architectural role for a nuclear noncoding RNA: NEAT1 RNA is essential for the structure of paraspeckles. Mol. Cell.

[21] Liang J., Liu C., Xu D., Xie K., Li A. (2022). LncRNA NEAT1 facilitates glioma progression via stabilizing PGK1. J. Transl. Med..

[22] Huang G., He X., Wei X. (2018). lncRNA NEAT1 promotes cell proliferation and invasion by regulating miR-365/RGS20 in oral squamous cell carcinoma. Oncol. Rep..

[23] Wang P., Wu T., Zhou H., Jin Q., He G., Yu H., Xuan L., Wang X., Tian L., Sun Y. (2016). Long noncoding RNA NEAT1 promotes laryngeal squamous cell cancer through regulating miR-107/CDK6 pathway. J. Exp. Clin. Cancer Res..

[24] Liu F., Tai Y., Ma J. (2018). LncRNA NEAT1/let-7a-5p axis regulates the cisplatin resistance in nasopharyngeal carcinoma by targeting Rsf-1 and modulating the Ras-MAPK pathway. Cancer Biol. Ther..

[25] Wang J., Zhang J., Liu H., Meng L., Gao X., Zhao Y., Wang C., Gao X., Fan A., Cao T. (2024). N6-methyladenosine reader hnRNPA2B1 recognizes and stabilizes NEAT1 to confer chemoresistance in gastric cancer. Cancer Commun..

[26] Zhuang S.T., Cai Y.J., Liu H.P., Qin Y., Wen J.F. (2020). LncRNA NEAT1/miR-185-5p/IGF2 axis regulates the invasion and migration of colon cancer. Mol. Genet. Genom. Med..

[27] Luo J., Xie K., Gao X., Yao Y., Wang G., Shao C., Li X., Xu Y., Ren B., Hu L. (2020). Long Noncoding RNA Nuclear Paraspeckle Assembly Transcript 1 Promotes Progression and Angiogenesis of Esophageal Squamous Cell Carcinoma Through miR-590-3p/MDM2 Axis. Front. Oncol..

[28] Wang Z., Zou Q., Song M., Chen J. (2017). NEAT1 promotes cell proliferation and invasion in hepatocellular carcinoma by negative regulating miR-613 expression. Biomed. Pharmacother..

[29] Gu J., Wang Q., Mo J., Qin T., Qian W., Duan W., Han L., Wang Z., Ma Q., Ma J. (2025). NEAT1 promotes the perineural invasion of pancreatic cancer via the E2F1/GDNF axis. Cancer Lett..

[30] Zhen S., Jia Y., Zhao Y., Wang J., Zheng B., Liu T., Duan Y., Lv W., Wang J., Xu F. (2024). NEAT1_1 confers gefitinib resistance in lung adenocarcinoma through promoting AKR1C1-mediated ferroptosis defence. Cell Death Discov..

[31] Wen J., Zheng W., Zeng L., Wang B., Chen D., Chen Y., Lu X., Shao C., Chen J., Fan M. (2023). LTF Induces Radioresistance by Promoting Autophagy and Forms an AMPK/SP2/NEAT1/miR-214-5p Feedback Loop in Lung Squamous Cell Carcinoma. Int. J. Biol. Sci..

[32] Zang F., Rao Y., Zhu X., Wu Z., Jiang H. (2020). Shikonin suppresses NEAT1 and Akt signaling in treating paclitaxel-resistant non-small cell of lung cancer. Mol. Med..

[33] Liu T., Wang H., Fu Z., Wang Z., Wang J., Gan X., Wang A., Wang L. (2022). Methyltransferase-like 14 suppresses growth and metastasis of renal cell carcinoma by decreasing long noncoding RNA NEAT1. Cancer Sci..

[34] Li K., Niu L., Zhang X., Li T., Zhou X., Wang L., Han J., Wang Z. (2025). NEAT1 is a therapeutic target for reversing T-cell exhaustion in bladder cancer. J. Immunother. Cancer.

[35] Wen S., Wei Y., Zen C., Xiong W., Niu Y., Zhao Y. (2020). Long non-coding RNA NEAT1 promotes bone metastasis of prostate cancer through N6-methyladenosine. Mol. Cancer.

[36] Xu D., Dong P., Xiong Y., Yue J., Konno Y., Ihira K., Kobayashi N., Todo Y., Watari H. (2020). MicroRNA-361-Mediated Inhibition of HSP90 Expression and EMT in Cervical Cancer Is Counteracted by Oncogenic lncRNA NEAT1. Cells.

[37] Luo X., Wei Q., Jiang X., Chen N., Zuo X., Zhao H., Liu Y., Liu X., Xie L., Yang Y. (2024). CSTF3 contributes to platinum resistance in ovarian cancer through alternative polyadenylation of lncRNA NEAT1 and generating the short isoform NEAT1_1. Cell Death Dis..

[38] Dong P., Xiong Y., Yue J., Xu D., Ihira K., Konno Y., Kobayashi N., Todo Y., Watari H. (2019). Long noncoding RNA NEAT1 drives aggressive endometrial cancer progression via miR-361-regulated networks involving STAT3 and tumor microenvironment-related genes. J. Exp. Clin. Cancer Res..

[39] Li J.H., Zhang S.Q., Qiu X.G., Zhang S.J., Zheng S.H., Zhang D.H. (2017). Long non-coding RNA NEAT1 promotes malignant progression of thyroid carcinoma by regulating miRNA-214. Int. J. Oncol..

[40] Park M.K., Zhang L., Min K.W., Cho J.H., Yeh C.C., Moon H., Hormaechea-Agulla D., Mun H., Ko S., Lee J.W. (2021). NEAT1 is essential for metabolic changes that promote breast cancer growth and metastasis. Cell Metab..

[41] Luan L., Hu Q., Wang Y., Lu L., Ling J. (2021). Knockdown of lncRNA NEAT1 expression inhibits cell migration, invasion and EMT by regulating the miR-24-3p/LRG1 axis in retinoblastoma cells. Exp. Ther. Med..

[42] Xia Y., Zhou Y., Han H., Li P., Wei W., Lin N. (2019). lncRNA NEAT1 facilitates melanoma cell proliferation, migration, and invasion via regulating miR-495-3p and E2F3. J. Cell. Physiol..

[43] Alharthi N.S., Al-Zahrani M.H., Hazazi A., Alhuthali H.M., Gharib A.F., Alzahrani S., Altalhi W., Almalki W.H., Khan F.R. (2024). Exploring the lncRNA-VEGF axis: Implications for cancer detection and therapy. Pathol. Res. Pract..

[44] Ji A., Li H., Fu X., Zhang Y., Liu Y. (2024). Long non-coding RNA NEAT1 induced by BHLHE40 activates Wnt/beta-catenin signaling and potentiates colorectal cancer progression. Cell Div..

[45] Wang Q.M., Lian G.Y., Sheng S.M., Xu J., Ye L.L., Min C., Guo S.F. (2024). Exosomal lncRNA NEAT1 Inhibits NK-Cell Activity to Promote Multiple Myeloma Cell Immune Escape via an EZH2/PBX1 Axis. Mol. Cancer Res..

[46] Chen W., Li Y., Zhou Q., Peng W., Cao M., Zhao Y., Yang Z., Xiong S., Huang H., Liu L. (2025). The cancer-associated fibroblast facilitates YAP liquid-liquid phase separation to promote cancer cell stemness in HCC. Cell Commun. Signal..

[47] Zhang X., Kang Z., Xie X., Qiao W., Zhang L., Gong Z., Chen Y., Shen W. (2020). Silencing of HIF-1alpha inhibited the expression of lncRNA NEAT1 to suppress development of hepatocellular carcinoma under hypoxia. Am. J. Transl. Res..

[48] Kuriakose B.B., Hjazi A., Saleh R.O., Bishoyi A.K., Jyothi S.R., Almalki S.G., Sridevi G., Chaudhary K., Zwamel A.H., Matchonov O. (2025). LncRNAs in hypoxic microenvironment; insight in their impact in cancer biology. Funct. Integr. Genom..

[49] Guo L., Tang Y., Wang Y., Xu H. (2021). Prognostic Value of lncRNA NEAT1 as a New Biomarker in Digestive System Tumors: A Systematic Study and Meta-analysis. Expert Rev. Mol. Diagn..

[50] Shi W., Zhang Z., Xu X., Tian Y., Feng L., Huang X., Du Y., Li Z. (2025). Single-cell and spatial transcriptomics integration: New frontiers in tumor microenvironment and cellular communication. Front. Immunol..

[51] Kang D.H., Lee J., Im S., Chung C. (2024). Navigating the Complexity of Resistance in Lung Cancer Therapy: Mechanisms, Organoid Models, and Strategies for Overcoming Treatment Failure. Cancers.

[52] Marcu L.G., Marcu D.C., Costin I.C., Zahu R., Straciuc O. (2025). From immunohistochemistry to machine learning-based patient stratification by tumour proliferation characteristics in head and neck cancer. Crit. Rev. Oncol./Hematol..

[53] De Domenico S., La Banca V., D’Amico S., Nicolai S., Peschiaroli A. (2025). Defining the transcriptional routes controlling lncRNA NEAT1 expression: Implications in cellular stress response, inflammation, and differentiation. Discov. Oncol..

[54] Brown J.A., Valenstein M.L., Yario T.A., Tycowski K.T., Steitz J.A. (2012). Formation of triple-helical structures by the 3′-end sequences of MALAT1 and MENbeta noncoding RNAs. Proc. Natl. Acad. Sci. USA.

[55] Li R., Harvey A.R., Hodgetts S.I., Fox A.H. (2017). Functional dissection of NEAT1 using genome editing reveals substantial localization of the NEAT1_1 isoform outside paraspeckles. RNA.

[56] Zakutansky P.M., Ku L., Zhang G., Shi L., Li Y., Yao B., Bassell G.J., Read R.D., Feng Y. (2024). Isoform balance of the long noncoding RNA NEAT1 is regulated by the RNA-binding protein QKI, governs the glioma transcriptome, and impacts cell migration. J. Biol. Chem..

[57] Knutsen E., Harris A.L., Perander M. (2022). Expression and functions of long non-coding RNA NEAT1 and isoforms in breast cancer. Br. J. Cancer.

[58] Yamazaki T., Souquere S., Chujo T., Kobelke S., Chong Y.S., Fox A.H., Bond C.S., Nakagawa S., Pierron G., Hirose T. (2018). Functional Domains of NEAT1 Architectural lncRNA Induce Paraspeckle Assembly through Phase Separation. Mol. Cell.

[59] Knott G.J., Bond C.S., Fox A.H. (2016). The DBHS proteins SFPQ, NONO and PSPC1: A multipurpose molecular scaffold. Nucleic Acids Res..

[60] Zhang M., Weng W., Zhang Q., Wu Y., Ni S., Tan C., Xu M., Sun H., Liu C., Wei P. (2018). The lncRNA NEAT1 activates Wnt/beta-catenin signaling and promotes colorectal cancer progression via interacting with DDX5. J. Hematol. Oncol..

[61] Wu D., Li H., Wang J., Li H., Xiao Q., Zhao X., Huo Z. (2020). LncRNA NEAT1 promotes gastric cancer progression via miR-1294/AKT1 axis. Open Med..

[62] Liang J., Fang F., Gao X., Shi J., Zhao J., Zhao Y. (2024). LncRNA NEAT1 promotes proliferation, migration, and invasion of laryngeal squamous cell carcinoma cells through miR-411-3p/FZD3-mediated Wnt signaling pathway. BMC Cancer.

[63] Lu Y., Li T., Wei G., Liu L., Chen Q., Xu L., Zhang K., Zeng D., Liao R. (2016). The long non-coding RNA NEAT1 regulates epithelial to mesenchymal transition and radioresistance in through miR-204/ZEB1 axis in nasopharyngeal carcinoma. Tumour Biol..

[64] Glaviano A., Foo A.S.C., Lam H.Y., Yap K.C.H., Jacot W., Jones R.H., Eng H., Nair M.G., Makvandi P., Geoerger B. (2023). PI3K/AKT/mTOR signaling transduction pathway and targeted therapies in cancer. Mol. Cancer.

[65] Spring L., Bardia A., Modi S. (2016). Targeting the cyclin D-cyclin-dependent kinase (CDK) 4/6-retinoblastoma pathway with selective CDK 4/6 inhibitors in hormone receptor-positive breast cancer: Rationale, current status, and future directions. Discov. Med..

[66] Raha P., Thomas S., Thurn K.T., Park J., Munster P.N. (2015). Combined histone deacetylase inhibition and tamoxifen induces apoptosis in tamoxifen-resistant breast cancer models, by reversing Bcl-2 overexpression. Breast Cancer Res..

[67] Lees A., Sessler T., McDade S. (2021). Dying to Survive-The p53 Paradox. Cancers.

[68] Elmi A., Makvandi M., Weng C.C., Hou C., Clark A.S., Mach R.H., Mankoff D.A. (2019). Cell-Proliferation Imaging for Monitoring Response to CDK4/6 Inhibition Combined with Endocrine-Therapy in Breast Cancer: Comparison of [(18)F]FLT and [(18)F]ISO-1 PET/CT. Clin. Cancer Res..

[69] Muhammad S., Jiang Z., Liu Z., Kaur K., Wang X. (2013). The role of EGFR monoclonal antibodies (MoABs) cetuximab/panitumab, and BRAF inhibitors in BRAF mutated colorectal cancer. J. Gastrointest. Oncol..

[70] Kanakaveti V., Ramasamy S., Kanumuri R., Balasubramanian V., Saravanan R., Ezhil I., Pitani R., Venkatraman G., Rayala S.K., Gromiha M.M. (2022). Novel BH4-BCL-2 Domain Antagonists Induce BCL-2-Mediated Apoptosis in Triple-Negative Breast Cancer. Cancers.

[71] Hsu P.C., Tsai C.C., Lin Y.H., Kuo C.Y. (2025). Therapeutic Targeting of Apoptosis, Autophagic Cell Death, Necroptosis, Pyroptosis, and Ferroptosis Pathways in Oral Squamous Cell Carcinoma: Molecular Mechanisms and Potential Strategies. Biomedicines.

[72] Zhu J., Petit P., Van den Eynde B.J. (2019). Apoptosis of tumor-infiltrating T lymphocytes: A new immune checkpoint mechanism. Cancer Immunol. Immunother..

[73] Lv K.J., Yu S.Z., Wang Y., Zhang S.R., Li W.Y., Hou J., Tan D.L., Guo H., Hou Y.Z. (2024). Cancer-associated fibroblasts promote the progression and chemoresistance of HCC by inducing IGF-1. Cell Signal..

[74] Piezzo M., Cocco S., Caputo R., Cianniello D., Gioia G.D., Lauro V.D., Fusco G., Martinelli C., Nuzzo F., Pensabene M. (2020). Targeting Cell Cycle in Breast Cancer: CDK4/6 Inhibitors. Int. J. Mol. Sci..

[75] Jullien M., Gomez-Bougie P., Chiron D., Touzeau C. (2020). Restoring Apoptosis with BH3 Mimetics in Mature B-Cell Malignancies. Cells.

[76] Ji Y., Wang M., Li X., Cui F. (2019). The Long Noncoding RNA NEAT1 Targets miR-34a-5p and Drives Nasopharyngeal Carcinoma Progression via Wnt/beta-Catenin Signaling. Yonsei Med. J..

[77] Liu X., Shang W., Zheng F. (2018). Long non-coding RNA NEAT1 promotes migration and invasion of oral squamous cell carcinoma cells by sponging microRNA-365. Exp. Ther. Med..

[78] Lu H., Kang F. (2020). Down-regulating NEAT1 inhibited the viability and vasculogenic mimicry formation of sinonasal squamous cell carcinoma cells via miR-195-5p/VEGFA axis. Biosci. Rep..

[79] Liu Y.C., Liu S.Y., Lin Y.C., Liu C.J., Chang K.W., Lin S.C. (2024). The disruption of NEAT1-miR-125b-5p-SLC1A5 cascade defines the oncogenicity and differential immune profile in head and neck squamous cell carcinoma. Cell Death Discov..

[80] Xue F., Cheng Y., Xu L., Tian C., Jiao H., Wang R., Gao X. (2020). LncRNA NEAT1/miR-129/Bcl-2 signaling axis contributes to HDAC inhibitor tolerance in nasopharyngeal cancer. Aging.

[81] Arutyunyan I., Jumaniyazova E., Makarov A., Fatkhudinov T. (2023). In Vitro Models of Head and Neck Cancer: From Primitive to Most Advanced. J. Pers. Med..

[82] González-González R., Ortiz-Sarabia G., Molina-Frechero N., Salas-Pacheco J.M., Salas-Pacheco S.M., Lavalle-Carrasco J., López-Verdín S., Tremillo-Maldonado O., Bologna-Molina R. (2021). Epithelial-Mesenchymal Transition Associated with Head and Neck Squamous Cell Carcinomas: A Review. Cancers.

[83] Chen G., Sun J., Xie M., Yu S., Tang Q., Chen L. (2021). PLAU Promotes Cell Proliferation and Epithelial-Mesenchymal Transition in Head and Neck Squamous Cell Carcinoma. Front. Genet..

[84] Li H., Liu Y.T., Chen L., Zhou J.J., Chen D.R., Li S.J., Sun Z.J. (2021). CMTM4 regulates epithelial-mesenchymal transition and PD-L1 expression in head and neck squamous cell carcinoma. Mol. Carcinog..

[85] Liang F., Wang R., Du Q., Zhu S. (2021). An Epithelial-Mesenchymal Transition Hallmark Gene-Based Risk Score System in Head and Neck Squamous-Cell Carcinoma. Int. J. Gen. Med..

[86] Zhang M.J., Liu J., Wan S.C., Li J.X., Wang S., Fidele N.B., Huang C.F., Sun Z.J. (2023). CSRP2 promotes cell stemness in head and neck squamous cell carcinoma. Head Neck.

[87] Elmusrati A., Wang J., Wang C. (2021). Tumor microenvironment and immune evasion in head and neck squamous cell carcinoma. Int. J. Oral Sci..

[88] Pawlicka M., Gumbarewicz E., Błaszczak E., Stepulak A. (2024). Transcription Factors and Markers Related to Epithelial-Mesenchymal Transition and Their Role in Resistance to Therapies in Head and Neck Cancers. Cancers.

[89] Liu R., Hu G., Li Y., Lu T., Pan M., Wang M., Cheng Z., Chen L. (2025). IGF2BP1 promotes the progression of head and neck squamous cell carcinoma by activating PI3K/AKT/mTOR signaling pathway and inducing epithelial-mesenchymal transition. World J. Surg. Oncol..

[90] He K., Zhu Z.B., Shu R., Hong A. (2020). LncRNA NEAT1 mediates progression of oral squamous cell carcinoma via VEGF-A and Notch signaling pathway. World J. Surg. Oncol..

[91] Sur S., Davray D., Basu S., Kheur S., Pal J.K., Nagar S., Sanap A., Rudagi B.M., Gupta S. (2024). Novel insights on oral squamous cell carcinoma management using long non-coding RNAs. Oncol. Res..

[92] Cohen E.R., Misztal C., Dable C., Gomez-Fernandez C., Bhatia R.G., Roth P., Ma R., Trosman S., Green C., Nicolli E. (2022). Redefining Perineural Invasion in Head and Neck Cutaneous Squamous Cell Carcinoma. Otolaryngol. Neck Surg..

[93] Zhou J., Liu C., Amornphimoltham P., Cheong S.C., Gutkind J.S., Chen Q., Wang Z. (2024). Mouse Models for Head and Neck Squamous Cell Carcinoma. J. Dent. Res..

[94] Zou Y., Duan H., Deng Z., Xiang R., Zhao J., Zhang Z., Hu W., Yang Y., Yan Z., Wen S. (2025). Single-cell atlas profiling revealed cellular characteristics and dynamic changes after PD-1 blockade therapy of brain metastases from laryngeal squamous cell carcinoma. Mol. Cell. Biochem..

[95] Fresno Vara J.A., Casado E., de Castro J., Cejas P., Belda-Iniesta C., González-Barón M. (2004). PI3K/Akt signalling pathway and cancer. Cancer Treat. Rev..

[96] Bahar M.E., Kim H.J., Kim D.R. (2023). Targeting the RAS/RAF/MAPK pathway for cancer therapy: From mechanism to clinical studies. Signal Transduct. Target. Ther..

[97] Liu J., Xiao Q., Xiao J., Niu C., Li Y., Zhang X., Zhou Z., Shu G., Yin G. (2022). Wnt/beta-catenin signalling: Function, biological mechanisms, and therapeutic opportunities. Signal Transduct. Target. Ther..

[98] Zhou B., Lin W., Long Y., Yang Y., Zhang H., Wu K., Chu Q. (2022). Notch signaling pathway: Architecture, disease, and therapeutics. Signal Transduct. Target. Ther..

[99] Zhang Y. (2024). LncRNA-encoded peptides in cancer. J. Hematol. Oncol..

[100] Yang C., Li Z., Li Y., Xu R., Wang Y., Tian Y., Chen W. (2017). Long non-coding RNA NEAT1 overexpression is associated with poor prognosis in cancer patients: A systematic review and meta-analysis. Oncotarget.

[101] Lin N.C., Hsia S.M., Wang T.H., Li P.J., Tseng Y.H., Chiu K.C., Tu H.F., Shih Y.H., Shieh T.M. (2022). The relation between NEAT1 expression level and survival rate in patients with oral squamous cell carcinoma. J. Dent. Sci..

[102] Zhu L., He Y., Feng G., Yu Y., Wang R., Chen N., Yuan H. (2021). Genetic variants in long non-coding RNAs UCA1 and NEAT1 were associated with the prognosis of oral squamous cell carcinoma. Int. J. Oral Maxillofac. Surg..

[103] Dong P., Xiong Y., Yue J., Hanley S.J.B., Kobayashi N., Todo Y., Watari H. (2018). Long Non-coding RNA NEAT1: A Novel Target for Diagnosis and Therapy in Human Tumors. Front. Genet..

[104] Liu Z., Wu K., Wu J., Tian D., Chen Y., Yang Z., Wu A. (2019). NEAT1 is a potential prognostic biomarker for patients with nasopharyngeal carcinoma. J. Cell. Biochem..

[105] Wang P., Li Q.Y., Sun Y.N., Wang J.T., Liu M. (2021). Long Noncoding RNA NEAT1: A Potential Biomarker in the Progression of Laryngeal Squamous Cell Carcinoma. ORL.

[106] Lin N.C., Hsia S.M., Vu Nguyen T.H., Wang T.H., Sun K.T., Chiu K.C., Shih Y.H., Shieh T.M. (2024). The association between the expression level of nuclear paraspeckle assembly transcript 1 and the survival rate of head and neck cancer patients after treatment. J. Dent. Sci..

[107] Song M., Bai H., Zhang P., Zhou X., Ying B. (2023). Promising applications of human-derived saliva biomarker testing in clinical diagnostics. Int. J. Oral Sci..

[108] Zhang Y., Lun L., Li H., Wang Q., Lin J., Tian R., Pan H., Zhang H., Chen X. (2017). The Value of lncRNA NEAT1 as a Prognostic Factor for Survival of Cancer Outcome: A Meta-Analysis. Sci. Rep..

[109] Barham W.T., Stagg M.P., Mualla R., DiLeo M., Kansara S. (2025). Recurrent and Metastatic Head and Neck Cancer: Mechanisms of Treatment Failure, Treatment Paradigms, and New Horizons. Cancers.

[110] Li Q., Tie Y., Alu A., Ma X., Shi H. (2023). Targeted therapy for head and neck cancer: Signaling pathways and clinical studies. Signal Transduct. Target. Ther..

[111] Cui L., Lu Y., Zheng J., Guo B., Zhao X. (2023). ACTN1 promotes HNSCC tumorigenesis and cisplatin resistance by enhancing MYH9-dependent degradation of GSK-3beta and integrin beta1-mediated phosphorylation of FAK. J. Exp. Clin. Cancer Res..

[112] Rahimi S.M., Bagheri A. (2025). Non-coding RNAs’ pivotal importance in modulation of cancer sensitivity to Topotecan: A systematic review. Med. Oncol..

[113] Sun Q., Lei X., Yang X. (2025). The crosstalk between non-coding RNAs and oxidative stress in cancer progression. Genes Dis..

[114] Xiao Y., Ran R., Zhu C., Ji Y., Shi J., Ye R., Geng C., Li R., Tang G., Wang W. (2025). Layered double hydroxide-delivered si-NEAT1 inhibits breast cancer immune evasion and malignant progression by modulating the miR-141-3p/PD-L1 axis. Bioorganic Chem..

[115] Tsuchiya H., Hanaki T., Sakabe T., Tokuyasu N., Nagahara T., Umekita Y., Isomoto H., Fujiwara Y., Nanba D. (2025). Immune evasion from macrophages by NEAT1-induced CD24 in liver cancer. Oncogene.

[116] Chen D., Wang J., Li Y., Xu C., Fanzheng M., Zhang P., Liu L. (2023). LncRNA NEAT1 suppresses cellular senescence in hepatocellular carcinoma via KIF11-dependent repression of CDKN2A. Clin. Transl. Med..

[117] Abdellatif A.A.H., Scagnetti G., Younis M.A., Bouazzaoui A., Tawfeek H.M., Aldosari B.N., Almurshedi A.S., Alsharidah M., Rugaie O.A., Davies M.P.A. (2023). Non-coding RNA-directed therapeutics in lung cancer: Delivery technologies and clinical applications. Colloids Surf. B Biointerfaces.

[118] Kukharsky M.S., Ninkina N.N., An H., Telezhkin V., Wei W., Meritens C.R., Cooper-Knock J., Nakagawa S., Hirose T., Buchman V.L. (2020). Long non-coding RNA Neat1 regulates adaptive behavioural response to stress in mice. Transl. Psychiatry.

[119] Kozłowska J., Kozioł K., Stasiak M., Obacz J., Guglas K., Poter P., Mackiewicz A., Kolenda T. (2020). The role of NEAT1 lncRNA in squamous cell carcinoma of the head and neck is still difficult to define. Contemp. Oncol..

[120] Vishnubalaji R., Elango R., Alajez N.M. (2022). LncRNA-Based Classification of Triple Negative Breast Cancer Revealed Inherent Tumor Heterogeneity and Vulnerabilities. Noncoding RNA.

[121] Mu D., Han B., Huang H., Zheng Y., Zhang J., Shi Y. (2025). Unraveling the advances of non-coding RNAs on the tumor microenvironment: Innovative strategies for cancer therapies. J. Transl. Med..

[122] Zhang D., Pei S., Feng Z., Xia G. (2025). Functions and mechanisms of lncRNAs in immune escape and their application in immunotherapy for colorectal cancer. J. Transl. Med..

[123] Garbo E., Del Rio B., Ferrari G., Cani M., Napoli V.M., Bertaglia V., Capelletto E., Rolfo C., Novello S., Passiglia F. (2023). Exploring the Potential of Non-Coding RNAs as Liquid Biopsy Biomarkers for Lung Cancer Screening: A Literature Review. Cancers.

[124] Rettig E.M., Chung C.H., Bishop J.A., Howard J.D., Sharma R., Li R.J., Douville C., Karchin R., Izumchenko E., Sidransky D. (2015). Cleaved NOTCH1 Expression Pattern in Head and Neck Squamous Cell Carcinoma Is Associated with NOTCH1 Mutation, HPV Status, and High-Risk Features. Cancer Prev. Res..

[125] Idogawa M., Ohashi T., Sasaki Y., Nakase H., Tokino T. (2017). Long non-coding RNA NEAT1 is a transcriptional target of p53 and modulates p53-induced transactivation and tumor-suppressor function. Int. J. Cancer.

[126] Hayrapetyan L., Roth S.M., Quintin A., Hovhannisyan L., Medo M., Riedo R., Ott J.G., Albers J., Aebersold D.M., Zimmer Y. (2025). HPV and p53 Status as Precision Determinants of Head and Neck Cancer Response to DNA-PKcs Inhibition in Combination with Irradiation. Mol. Cancer Ther..

[127] Brennan J.A., Boyle J.O., Koch W.M., Goodman S.N., Hruban R.H., Eby Y.J., Couch M.J., Forastiere A.A., Sidransky D. (1995). Association between cigarette smoking and mutation of the p53 gene in squamous-cell carcinoma of the head and neck. N. Engl. J. Med..

[128] Idogawa M., Nakase H., Sasaki Y., Tokino T. (2019). Prognostic Effect of Long Noncoding RNA NEAT1 Expression Depends on p53 Mutation Status in Cancer. J. Oncol..

[129] Li X., Wang X., Song W., Xu H., Huang R., Wang Y., Zhao W., Xiao Z., Yang X. (2018). Oncogenic Properties of NEAT1 in Prostate Cancer Cells Depend on the CDC5L-AGRN Transcriptional Regulation Circuit. Cancer Res..

[130] Zhang J., Guo S., Piao H.Y., Wang Y., Wu Y., Meng X.Y., Yang D., Zheng Z.C., Zhao Y. (2019). ALKBH5 promotes invasion and metastasis of gastric cancer by decreasing methylation of the lncRNA NEAT1. J. Physiol. Biochem..

[131] Bhattacharya A., Wang K., Penailillo J., Chan C.N., Fushimi A., Yamashita N., Daimon T., Haratake N., Ozawa H., Nakashoji A. (2024). MUC1-C regulates NEAT1 lncRNA expression and paraspeckle formation in cancer progression. Oncogene.

[132] Yan K., Fu Y., Zhu N., Wang Z., Hong J.L., Li Y., Li W.J., Zhang H.B., Song J.H. (2019). Repression of lncRNA NEAT1 enhances the antitumor activity of CD8(+)T cells against hepatocellular carcinoma via regulating miR-155/Tim-3. Int. J. Biochem. Cell Biol..

[133] Meng H., Chen Z., Han M., Cheng Q., Shang Y., Wang H., Lu S. (2025). Functional mechanism and clinical implications of lncRNA NEAT1 in traumatic fractures and their correlation with delayed healing. J. Orthop. Surg. Res..

[134] Wang J., Tao X., Zhu J., Dai Z., Du Y., Xie Y., Chu X., Fu G., Lei Z. (2025). Tumor organoid-immune co-culture models: Exploring a new perspective of tumor immunity. Cell Death Discov..

[135] Mazurek M., Brzozowska A., Małecka-Massalska T., Powrózek T. (2025). Plasma Circulating lncRNAs: MALAT1 and NEAT1 as Biomarkers of Radiation-Induced Adverse Effects in Laryngeal Cancer Patients. Diagnostics.

[136] Lo P., Wolfson B., Zhou Q. (2016). Cellular, physiological and pathological aspects of the long non-coding RNA NEAT1. Front. Biol..

[137] Li C., Guo H., Zhai P., Yan M., Liu C., Wang X., Shi C., Li J., Tong T., Zhang Z. (2024). Spatial and Single-Cell Transcriptomics Reveal a Cancer-Associated Fibroblast Subset in HNSCC That Restricts Infiltration and Antitumor Activity of CD8+ T Cells. Cancer Res..

[138] Zhao N., Zhang J., Sun T., Zhang X., Liu J., Yu H., Zhang H. (2025). AI-driven multi-omics integration of cancer-associated fibroblasts for prognostic modeling and therapeutic target discovery in head and neck squamous cell carcinoma. npj Precis. Oncol..

[139] You C., Park J., Jang J.Y., Noh J., Lee J., Kwon G., Yu M.S., Chung Y.S., Lee S.J., Kang K. (2026). Single-Nucleus Multi-Omics Reveals Hypoxia-Driven Angiogenic Programs and Their Epigenetic Control in Sinonasal Squamous Cell Carcinoma. Adv. Sci..

[140] Wang Q., Meng Y., Liu Y., Zhang S., Wang Y., Chen P., Xiang B., Zhou M., Gong Z., Zeng Z. (2025). Arecoline upregulates CD155 expression to facilitate immune evasion in oral squamous cell carcinoma. J. Immunother. Cancer.

[141] Li W., Nishino M., Reed E., Akshinthala D., Pasha H.A., Anderson E.S., Huang L., Hebestreit H., Monti S., Gomez E.D. (2025). Head and neck tumor organoid grown under simplified media conditions model tumor biology and chemoradiation responses. Sci. Rep..

[142] Alabi R.O., Guntinas-Lichius O., Elmusrati M., Almangush A., Tiblom Ehrsson Y., Laurell G., Mäkitie A.A. (2025). Machine learning for survival outcome in head and neck squamous cell carcinoma: A multicenter validation study. Sci. Rep..

[143] Gong T., Lao I.W., Chio C.F., Zhang X., Ren M., Li W., Qu N., Ni X., Gong T., Gu Y. (2026). Machine learning-driven proteomics classifier deciphers tumor origins of primary and metastatic squamous cell carcinomas. Biomark. Res..

[144] Jiang S., Li H., Zhang L., Mu W., Zhang Y., Chen T., Wu J., Tang H., Zheng S., Liu Y. (2025). Generic Diagramming Platform (GDP): A comprehensive database of high-quality biomedical graphics. Nucleic Acids Res..

